# 多原发肺癌诊疗策略的最新进展

**DOI:** 10.3779/j.issn.1009-3419.2023.102.44

**Published:** 2023-11-20

**Authors:** Bangsheng LI, Zhenghong YANG, Yingding ZHAO, Ying CHEN, Yunchao HUANG

**Affiliations:** 650118 昆明，昆明医科大学第三附属医院胸外一科; Department of Thoracic Surgery I, Third Affiliated Hospital of Kunming Medical University, Kunming 650118, China

**Keywords:** 肺肿瘤, 第二代测序技术, 手术, 放射治疗, 消融, 靶向治疗, 免疫治疗, Lung neoplasms, Next-generation sequencing, Surgery, Radiation therapy, Ablation, Targeted therapy, Immunotherapy

## Abstract

随着后疫情时代的到来，计算机断层扫描在肺癌筛查中运用愈加广泛，多原发性肺癌（multiple primary lung cancer, MPLC）的检出率在不同国家和地区也随之飙升。尽管先进的组织学和测序技术不断应用于该领域，但MPLC和肺内转移（intrapulmonary metastasis, IM）的鉴别仍具有挑战性。近年来，遗传和环境因素在MPLC中的特殊机制逐渐被人们发掘。在MPLC的治疗中，肺叶切除术仍然居于主要地位，但特定肿瘤亚肺叶切除并未对生存产生负面影响，这似乎是一种合理的选择。随着理念的革新，以手术为主的综合治疗是一种新趋势。其中，立体定向消融放疗（stereotactic ablative radiotherapy, SABR）和肺消融技术成为早期不可切除肿瘤和控制残留病灶的有效治疗手段。此外，驱动基因阳性的靶向治疗和免疫治疗在术后辅助阶段也显示出良好的结果。本文拟基于最新的研究成果对MPLC的管理进行综述。

## Abstract (en)

As the utilization of computed tomography in lung cancer screening becomes more prevalent in the post-pandemic era, the incidence of multiple primary lung cancer (MPLC) has surged in various countries and regions. Despite the continued application of advanced histologic and sequencing technologies in this research field, the differentiation between MPLC and intrapulmonary metastasis (IM) remains challenging. In recent years, the specific mechanisms of genetic and environmental factors in MPLC have gradually come to light. Lobectomy still predominates in the treatment of MPLC, but the observation that tumor-specific sublobar resection has not detrimentally impacted survival appears to be a viable option. With the evolution of paradigms, the amalgamated treatment, primarily surgical, is an emerging trend. Among these, stereotactic ablative radiotherapy (SABR) and lung ablation techniques have emerged as efficacious treatments for early unresectable tumors and control of residual lesions. Furthermore, targeted therapies for driver-positive mutations and immunotherapy have demonstrated promising outcomes in the postoperative adjuvant phase. In this manuscript, we intend to provide an overview of the management of MPLC based on the latest discoveries.

肺癌是中国乃至世界范围内发病率和死亡率最高的恶性肿瘤之一^[1,2]^。随着胸部计算机断层扫描（computed tomography, CT）的广泛运用和人均期望寿命的延长罹患多原发性肺癌（multiple primary lung cancer, MPLC）的概率增加^[3]^。临床数据^[4]^显示，MPLC的发病率在1%-20%之间，女性和非吸烟患者占比更高。但值得注意的是，自2019年新型冠状病毒肺炎（corona virus disease-19, COVID-19）全球大流行以来，接受肺部CT检查的人群较前明显增多，导致了肺部多发结节的快速增长^[5]^，但目前尚未对两者之间的关联性进行阐述。

Beyreuther^[6]^于1924年首次报道了1例MPLC，自此MPLC开始走入临床的视野。MPLC是指在肺内同时或先后发现2个或2个以上的原发性癌灶，这些癌灶之间在解剖上是相对分离的，且在起源上也是独立的^[7]^。为了更好地服务临床工作，根据两次诊断的间隔时间，将MPLC进一步划分为异时性多原发肺癌（metachronous multiple primary lung cancer, mMPLC）和同时性多原发肺癌（synchronous multiple primary lung cancer, sMPLC）^[8]^。在临床中，其诊断的关键在于与肺癌的肺内转移（intrapulmonary metastasis, IM）相鉴别^[9]^，本文主要从组织病理学、影像学和分子生物学3个方面总结二者的区别。

目前，许多MPLC患者被同时诊断出多个磨玻璃结节（ground glass nodules, GGNs），sMPLC患者的比例和5年生存率也在逐渐增加，而术后死亡率正在下降^[3]^，因此，手术是该疾病早期阶段的主要治疗方法^[10]^。同时，随着人均寿命的延长，一些肺癌患者会发展为mMPLC，重复手术可能导致该类患者肺功能严重受损，生活质量降低，因此，需要除手术外的有效干预来管理该患者群体^[11]^。

随着新技术和治疗手段的进步，特别是第二代测序技术（next-generation sequencing, NGS）的发展，MPLC的诊断和管理有了很大的改善。在本文中，我们回顾了截止2023年10月相关MPLC诊断和治疗的期刊文献，并总结了该领域的最新研究进展。

## 1 MPLC和IM的组织病理学差异

在肺部多发肿瘤中，MPLC和肺癌的IM的鉴别尤为重要，这直接影响到了准确分期和随后的临床管理。1975年，Martini和Melamed等^[7]^首次提出了MPLC的临床病理诊断标准（简称M-M标准），其主要内容包括：肿瘤是独立的，肿瘤病理类型不同，或肿瘤病理类型相同但至少位于不同的肺段、均起源于原位癌、无淋巴结转移和肺外转移。基于该诊断标准，以下4种情况仅依靠组织学来鉴别是可行的。首先，当患者病理组织类型不同时可以明确诊断^[7]^。其次，具有早期病变或原位癌成分的肿瘤，如鳞状细胞癌合并相邻鳞状原位癌，通常被认为是MPLC^[12]^。然而，在一些少数腺癌中，贴壁型生长模式可能代表侵袭性肿瘤，而不是早期病变^[13]^。因此，此类标准应该谨慎使用尤其是在有限区域内。第三，原位多灶性腺癌、微浸润性腺癌也通常被诊断为MPLC^[14]^。第四，最近的分子谱分析^[15]^表明，几乎所有独立的浸润性黏液腺癌是来自单个肿瘤的IM。M-M标准虽然是目前应用最广泛的诊断标准，但是，当病理学类型相同时，M-M标准对MPLC和IM的鉴别就缺乏准确性，且在临床中腺癌-腺癌组合是最常见的病理类型组合^[16]^。于是，2016年，国际肺癌研究协会（International Association for the Study of Lung Cancer, IASLC）^[17]^开始推广综合组织学评估（comprehensive histologic assessment, CHA）。近期，国际肺癌病理研究协会委员会也进行了该方法的效果评价^[18]^，结果显示其在评估MPLC方面具有良好的一致性，适用于大多数场景。

## 2 MPLC和IM的影像学区别

尽管MPLC和IM的准确鉴别涉及病理和基因组技术，但在治疗前区分两者仍然关键。如果术前MPLC被误诊为IM，患者可能会失去手术机会或受到不必要的化学和辐射损伤，此时强调治疗前的影像学证据变得非常重要^[19]^。目前，CT及正电子发射计算机断层显像（positron emission tomography/CT, PET/CT）仍然是鉴别MPLC和IM的主要影像学方法。临床上通过不同病灶的形态和PET/CT的SUVmax等指标综合诊断，MPLC在CT影像上以磨玻璃成分多见，而IM以实性成分为主且边缘光滑，少见毛刺或分叶征^[8]^。基于CT影像组学的研究验证了两者之间的形态区别，并与综合组织病理学的鉴别结果具有良好的一致性^[20,21]^。然而，值得注意的是，Li等^[22]^对多发GGNs的肺腺癌队列行全外显子测序时发现，部分GGNs存在同一克隆起源。SUVmax反映了肿瘤的代谢活性，与肿瘤组织学亚型相关。Liu等^[23]^发现sMPLC和IM的SUVmax比值存在显著差异，认为该比值有助于区分这两种肿瘤。为进一步提高MPLC的诊断准确率，Suh等^[24]^提出了一种结合临床和影像变量的算法，纳入指标包括CT上的病灶类型、病变形态、肿瘤的SUVmax以及是否存在N2/N3淋巴结转移和远处转移，结果显示，该模型的总体诊断准确性达88.9%，该结果对未来的临床推广是有利的。随着科技的发展，人工智能（artificial intelligence, AI）在诊断MPLC方面也越来越有效^[25]^。与之前的成像系统相比，AI可测量更多客观参数，如三维（3D）体积和可能的病理模式等，从而更准确地判定结节性质。尤其是在需要及时分期手术或定期随访的肺部多结节患者中，AI可以轻松展示多个结节的变化模式，并用客观数据展示视觉结果，从而减少临床医生耗时耗力地进行CT图像对比。对此，Li等^[26]^回顾了53例经过病理确诊的MPLC，并经过AI统计分析发现，AI的诊断准确率达88.8%。

## 3 基因组技术在诊断MPLC中的应用

目前常规的术前影像学和术后病理学评估不再能够可靠地区分MPLC与IM。在Chang等^[18]^的研究中，通过组织学评估与涵盖高达468个癌症相关基因的基因组检测进行对比分析，结果显示，22%的病例的前瞻性组织学预测与NGS不一致，尤其是对IM的预测，不一致率达44%。因此，采用NGS来补充组织学评估是有必要的。目前，靶向测序、单细胞测序、微阵列比较基因组杂交和NGS等分子技术已被运用于MPLC的特征分析^[27⇓-29]^。在NGS普及之前，包含一些致癌/抑癌基因（通常为1-5个基因）和MPLC中的染色体改变是重点，但低效的技术远远不足以分析MPLC基因组，阻碍了我们对MPLC基因组的深入了解^[30]^。NGS技术的出现，使基因组研究进入了一个新的高潮，为基于组织学评估的精准诊疗提供了新的思路和方向。

NGS是一种高通量测序技术，其通过同时对大量DNA或RNA分子进行并行测序，从而实现了更高效、快速地获取基因组或转录组的序列信息，其中，大基因测序panel、全基因组测序和染色体重排的应用更好地帮助我们区分了MPLC和IM^[30]^。Liu等^[31]^运用微阵列比较基因组杂交和全基因组测序技术分析了6例sMPLC患者的15个肺腺癌病灶和1例区域淋巴结转移的基因组谱，结果发现，在相同的体质遗传背景和环境暴露的背景下，同一个体的不同肺癌可能具有不同的基因组谱，并且可以由不同的分子事件驱动。同时，Li等^[32]^对154个亚实性结节样本进行全外显子组测序时发现，在亚实性结节之间存在显著的基因组异质性，多中心起源占主导地位，不同的突变谱强烈表明不同的克隆起源。然而，2018年，Li等^[22]^对2例患者的14个亚实性结节进行了综合NGS检测，结果显示，MPLC和IM在同一患者中共存，其中4个GGNs存在克隆相关性，这颠覆了我们对GGNs的印象，研究者认为这可能与肺癌的气腔播散相关。随后，为了最大程度地区分MPLC和IM，Chang等^[18]^使用大规模NGS panel进行分析，结果证明，表现出完全不重叠的独特突变的肿瘤可以被归为MPLC。相反，具有多个（≥2个）共突变的肿瘤被认为是IM。对于共享单个热点突变的肿瘤，MPLC和IM的判定需要检测额外的分子改变加以区分，例如同义突变和染色体臂水平增益和损失等。

其他基因组技术也在MPLC和IM的识别中发挥着重要作用。Liu等^[33]^对16例MPLC患者的40个肿瘤和匹配的血液样本进行了靶向测序，比较同一患者的不同肿瘤和不同患者肿瘤之间的突变谱时发现，12例患者的多个肿瘤中没有任何共同的突变，认为不同肿瘤之间重叠突变的分析有助于医生区分MPLC和IM^[33]^。此外，基因组技术在探索MPLC的起源和启动机制时也发挥了重要作用。Guo等^[34]^对MPLC患者肿瘤及癌旁组织细胞进行了单细胞测序，结果发现，上皮细胞的两种状态分别与免疫反应和细胞死亡相关，揭示了MPLC的细胞特征和细胞间连接，为MPLC的早期诊断和治疗提供了新的见解。总之，单纯地运用组织学评估或不全面的基因状态来诊断MPLC都是不可靠的。在影像和病理学证据的基础上，建议进一步采用广泛的NGS加以补充，以便在临床实践中对MPLC和IM进行强有力的区分。

## 4 MPLC的病因学

MPLC的病因学是一个复杂而多元化的话题，需要从多个角度进行研究和探索。数据^[35]^证明，在MPLC患者中烟草同样是主要的危险因素。流行病学监测和最终结果（Surveillance, Epidemiology, and End Results, SEER）数据库分析^[36]^表明，年龄较小、女性、早期阶段也是MPLC的危险因素。此外，越来越多的研究揭示了遗传因素与MPLC易感性之间的相关性，尤其是在非吸烟者和家族性肺癌中发挥着重要作用。Li等^[37]^在检测162例多原发家族肺癌患者的表皮生长因子受体（epidermal growth factor receptor, EGFR）基因时发现，EGFR种系突变等遗传变异可能是MPLC的驱动力。2021年，Chen等^[38]^首次证明微塑料暴露与肺部GGNs相关，将MPLC发病机制与环境因素联系起来。因此，遗传和特定的环境因素可能使MPLC患者具有独特的病理和分子特征，了解其对MPLC的影响并引入可能降低死亡率的准确建议至关重要^[39]^。

## 5 MPLC的外科治疗

### 5.1 手术的重要地位

随着外科术式和入路的创新和突破，衍生出了多种微创手术，包括：电视辅助胸腔镜手术（video-assisted thoracic surgery, VATS）和机器人辅助胸腔镜手术^[40]^。相较于传统开胸手术，胸腔镜手术明显提高了手术的精确性和可控性，较好地缩短住院时长，符合当下快速康复理念；同时，极大地缩小了手术创口，充分地增加了患者的依从性，尤其是在MPLC患者中^[41]^。目前，微创手术的发展是全球性的，其患者的生存率显著延长^[3]^。

### 5.2 肺叶切除的适用性

MPLC的管理应基于多学科团队讨论，更好地与IM相鉴别。在技术可行且不牺牲肿瘤学原则的前提下推荐胸腔镜手术路径。但MPLC的最佳手术策略尤其是针对第二结节的切除范围仍存在争议^[42]^。在过去的二十年中，大量研究一直致力于为MPLC寻找合适的治疗方案，并提供充足的临床证据。早在1995年，Ginsberg等^[43]^通过临床试验证明了肺叶切除术比亚肺叶切除术更具优越性。相继有研究者提出类似的观点，Aziz等^[44]^也建议第二结节行肺叶切除术+淋巴结清扫术。此外，在美国的一项大规模回顾性研究中^[45]^，对于IA期肺癌，进行亚肺叶切除会增加非R0切除的风险，减少淋巴结清扫的数量，从而影响N分期的升级，进而导致预后较差。但在微创治疗迅速发展以及大多数MPLC患者被同时检测出GGNs的背景下，迫切需要更优的手术方案来挽救肺功能，使患者长期获益^[46]^。

Kikunaga等^[47]^的研究数据显示，mMPLC全肺切除并未出现生存不良，亚肺叶切除的生存曲线相较于肺叶切除有下降趋势，应该采取更加积极的手术方式。2014年，关于93例sMPLC患者的研究结果^[48]^显示，亚肺叶切除是sMPLC预后的独立危险因素。然而，Kocaturk等^[49]^对26例sMPLC患者的术后生存进行了综合分析，接受全肺切除的患者存在生存不良趋势，而接受亚肺叶切除的患者中未观察到生存劣势。激进的手术方法可能会导致年龄较大或存在潜在合并症的患者的生存率降低或手术死亡率增加^[50]^。针对双侧MPLC患者的5年随访分析^[51]^发现，肺叶切除和亚肺叶切除的5年总生存（overall survival, OS）率没有显著差异。Watanabe等^[52]^回顾了85例sMPLC的生存结果和肺功能，sMPLC的肺段和楔形切除在保证治愈的同时可以更多地保留患者肺功能。Nomori等^[53]^的对肺叶切除与肺段切除进行倾向评分匹配比较发现，二者的无复发率、总生存率、术后并发症相似，进一步验证了亚肺叶切除的安全性。此外，2020年的一项荟萃分析^[54]^显示，MPLC进行肺叶切除和亚肺叶切除的OS相当，同时应避免全肺切除。基于现阶段的研究，早期MPLC患者在2010年之前的研究倾向于采用标准的肺叶切除。然而，在2010年之后的研究中，更多的关注被放在保留肺组织、提高远期生活质量方面^[55,56]^。然而，由于缺乏同期大量的临床研究，异质性较大以及多发病灶的实性占比和病理类型与预后息息相关等综合因素影响，目前的研究结果尚无法达成一致。

本文总结了MPLC切除方法和结果的研究，见表1。

**表1:** MPLC切除方法和结果的研究

Authors	Patient group	Study period	Major results	Study conclusions
Zuin A, et al.^[42]^	sMPLC+mMPLC: n=121 Initial surgery: Lobectomy: n=106; Sublobar: n=10; Pneumonectomy: n=5 Secondary surgery: Lobectomy: n=61; Sublobar: n=60	Jan 1995 to Dec 2010	5-year OS: Lobectomy: 57.5% Sublobar resection: 36% Pneumonectomy: 20%	Lobectomy should still be considered as the treatment of choice in the management of second primary lung cancer. However, lobectomy remains an effective option for high-risk patients with restricted pulmonary function. Completion pneumonectomy is associated with a negative long-term prognosis for survival
Kikunaga S, et al.^[47]^	sMPLC+mMPLC: n=239 sMPLC: Lobar-lobar: n=4; Lobar-sublobar: n=67; Sublobar-sublobar: n=51; Lobectomy: n=36 mMPLC: Lobar-lobar: n=13; Lobar-sublobar: n=44; Sublobar-sublobar: n=24	Jan 2010 to Dec 2018	5-year OS: sMPLC: 84.9% mMPLC: 75.2%	It was found that the survival rate showed a decreasing trend in the order of lobectomy, segmentectomy resection and wedge resection. For stage I cases, we could expect a good prognosis, and therefore surgical treatment should actively be performed
Ishikawa Y, et al.^[48]^	sMPLC: n=93 Bilateral lobectomies: n=10; Lobectomy+sublobar resection: n=27; Sublobar+sublobar resection: n=27; Pneumonectomy: n=1; Lobectomy: n=28	Apr 1995 to Dec 2009	5-year OS: Lobectomy: 92.5% Segmentectomy: 82.1% Wedge resection: 80.4%	The use of sublobar resection were independent predictors of poor survival. Lobectomy has a high cure rate and should be performed whenever possible
Kocaturk CI, et al.^[49]^	sMPLC: n=26 Pneumonectomy: n=11; Bilateral lobectomies: n=6; Lobectomy+sublobar resection: n=8; Sublobar resection: n=1	Jan 2001 to Dec 2008	5-year OS: Pneumonectomy: 27% No-pneumonectomy: 71.1%	Poor survival trend was observed in patients who received pneumonectomy
Jung EJ, et al.^[50]^	sMPLC: n=32 Pneumonectomy: n=5; Bilateral lobectomies+sublobar resection: n=1; Bilobectomy: n=4; Lobectomy+sublobar resection: n=14; Sublobar-sublobar: n=1; Lobectomy: n=7	Jan 1995 to Dec 2008	5-year OS: Pneumonectomy: 30% No-pneumonectomy: 73% Use of limited resection 5-year OS: 79.4% 5-year PFS: 74.5% Lobectomy or extended 5-year OS: 51.2% 5-year PFS: 34.2%	The decision of aggressive surgical treatments for sMPLC should be made carefully in the patients with old age and underlying comorbidities due to poor OS and increased surgical mortality
Tsunezuka Y, et al.^[51]^	BMPLC: n=53; sMPLC: n=10; mMPLC: n=43 Initial surgery: Lobectomy: n=35; Sublobar: n=18 Secondary surgery: Lobectomy: n=5; Sublobar: n=48	Jan 2007 to May 2019	5-year OS: Lobectomy: 78.7% Wedge or Segmentectomy: 67.9%	In the setting of BMPLC, there was no statistically significant difference in 5-year OS between lobectomy and sublobar resection (segmentectomy or wedge resection), while the latter approach allows for preservation of more pulmonary function in patients
Watanabe T, et al.^[52]^	sMPLC: n=85 Bilateral lobectomies: n=9; Lobectomy+sublobar resection: n=47; Sublobar resection: n=29	Jan 2010 to Sep 2020	5-year OS: All patients: 85.0% Patients in stage I: 94.9% Patients in ≥stage II: 62.6% (P<0.001)	Surgical treatment including limited resection, especially segmentectomy and wedge resection, for sMPLC can preserve pulmonary function while ensuring curability
Yang H, et al.^[55]^	sBMPLC: n=71; mBMPLC: n=30; Lobar-lobar: n=39; Lobar-sublobarr: n=49; Sublobar-sublobar: n=13	Jan 2001 to Jun 2014	5-year OS: Lobar-lobar: 70.6% Lobar-sublobar: 56.7% Sublobar-sublobar: 36.8%	The use of a limited resection procedure for the contralateral second nodule in cases with stage I BMPLC did not have a negative effect on the 5-year OS

MPLC: multiple primary lung cancer; sMPLC: synchronous multiple primary lung cancer; mMPLC: metachronous multiple primary lung cancer; BMPLC: bilateral multiple primary lung cancer; OS: overall survival; PFS: progression-free survival.

### 5.3 sMPLC和mMPLC的预后区别

Yang等^[55]^对101例MPLC患者的手术预后进行了分析，异时性双侧MPLC（metachronous bilateral MPLC, sBMPLC）和同时性双侧MPLC（synchronous bilateral MPLC, sBMPLC）之间的5年OS没有明显差异，这与之前的多项荟萃分析结果^[10,57]^一致。相比之下，2013年，对121例MPLC患者进行了生存分析，mMPLC患者的5年OS明显好于sMPLC患者^[42]^。因此，sMPLC和mMPLC患者预后差异也存在较大争议，尚需进一步深入研究来探讨其预后问题。

### 5.4 术后残留病灶的随访

系列临床研究^[58,59]^显示，20%-30%的GGNs病灶为主的早期肺癌切除时伴有多个侵入性较小的GGNs病灶，未来进展的比例在0%-40%之间。2015年，Shimada等^[46]^分析了存在多个GGNs病灶的肺癌患者的临床特点，初次手术后，39例患者总共有118个未切除的GGNs。其中，每个结节的生长频率为8%，23%出现了新的GGNs，但进展病灶和新病灶的出现均不影响生存。同一研究组于2020年将190例多灶性GGNs患者与孤立性肺腺癌患者进行了比较，发现多灶性GGNs腺癌和匹配的孤立性腺癌的无复发生存（recurrence-free survival, RFS）率，没有显著差异^[60]^。此外，单因素分析^[61]^显示，病灶的高实性成分占比是RFS较差的独立预测因素。因此，定期进行影像学检查和症状回顾的监测是值得推荐的。

## 6 以手术为主的综合治疗

### 6.1 放疗

立体定向消融放疗（stereotactic ablative radiotherapy, SABR）或立体定向放射治疗（stereotactic body radiotherapy, SBRT）被广泛用于治疗不可切除的I期非小细胞肺癌（non-small cell lung cancer, NSCLC）。SABR和SBRT均为高精度的放疗技术，均通过CT及磁共振成像（magnetic resonance imaging, MRI）来精确目标区域，可以将高剂量辐射安全地递送到较小的肿瘤中，而无需进行长期分级分割，二者在文献和临床中常互换使用^[62]^。Ball等^[63]^的III期临床对比实验（NCT01014130）发现，与常规放疗相比，SABR可以更好地控制原发灶，且不会增加主要毒性。此外，一项大样本回顾性研究^[64]^支持SBRT在MPLC患者中使用。为了更全面地评估I期NSCLC患者在接受SABR和手术治疗后的长期生存率，Chang等^[65]^进行了一项前瞻性研究，SABR后的长期生存率与接受手术治疗的患者相比并不逊色。然而，建议在确定管理方案之前行多学科讨论。

欧洲医学肿瘤学会（European Society for Medical Oncology, ESMO）临床实践指南建议MPLC应以治愈为目的进行评估，而放疗是一种有效的选择^[66]^。一项比较放疗与手术治疗首次接受肺癌根治术的MPLC患者的多中心回顾性研究^[67]^纳入了203例患者，分组治疗3年后的局部复发率在统计学上无明显差异。Shintani等^[68]^回顾了18例放疗联合手术治疗sMPLC的病例发现，为了尽可能保留MPLC患者的肺功能，SBRT是一种高效的局部治疗方法。此外，放疗的治疗模式呈现多样化趋势^[69]^。Chang等^[70]^在最近的II期临床实验（NCT03110978）中提出：SABR联合免疫治疗（immunotheray-SABR, I-SABR）。程序性细胞死亡受体1（programmed cell death 1, PD-1）抑制剂联合SABR治疗组患者的4年无事件生存（event-free suivival, EFS）率可达77%，较仅接受SABR治疗的患者高出24%，有望为早期NSCLC的系统性治疗提供高效且安全的新选择。总而言之，SABR已成为早期不可切除肺癌患者的首选方案，并初步验证了其在MPLC患者中的安全性和有效性。然而，关于放疗联合手术治疗MPLC的临床研究数据尚不充分。

### 6.2 肺消融技术的探索

消融治疗指通过消融针对局部肿瘤组织进行加热或冷冻，使肿瘤细胞发生不可逆损伤或凝固性坏死。肺消融治疗主要有3种：射频消融（radiofrequency ablation, RFA）、微波消融（microwave ablation, MWA）和冷冻消融（cryoablation, CA）。RFA和MWA利用热量导致肿瘤细胞坏死，而CA依赖于连续的冷冻和解冻^[71]^。MWA的优势在于能在较短的时间内产生较高的温度，且不受“热沉”效应的影响，但高功率也随之带来较高的手术风险，而RFA刚好与之相反。CA不会引起高温疼痛，对周围的组织影响较小，可以更安全地治疗靠近大血管的病灶；治疗过程中不产生电流或磁场，更适合植入心脏起搏器的患者，然而治疗时间长和消耗血小板是其弊端^[72]^。消融治疗的独特优势包括：可重复性强、同期或分期处理同侧/对侧多个病灶以及术后并发症少、经济效益较高等。随着手术理念和设备的不断创新发展，消融可单独或者联合手术同时进行。近些年，该技术已被较多地应用于MPLC的管理，并取得了令人满意的结果^[73,74]^。

Bao等^[75]^对15例手术联合MWA的MPLC患者进行了分析，在最大程度根治的情况下，充分保留了患者肺功能，无严重或死亡相关并发症。在VATS与MWA的对比研究^[76]^中发现，对于GGNs患者，两种治疗方式的3年OS率、局部无进展生存率相似，MWA可为GGNs患者提供替代治疗选择。2020年，在33例CT引导下经皮MWA治疗同步GGNs的病例中^[74]^，技术成功率100%，充分验证其安全性和有效性。此外，Wang等^[73]^对消融和手术治疗I期肺癌的临床结果进行了2年随访分析，两者的预后无明显差异（OS: 91.30% vs 90.59%），但住院时长和住院费用较肺叶切除有明显优势。随着肺癌早诊早筛的推进和人口老龄化的加剧，越来越多的GGNs被检出，且近25%的肺癌患者属于手术高风险^[77]^。同时，GGNs由于其生长特征，通常被认为是惰性腺癌亚型。因此，肺叶切除术可能不是GGNs样MPLC患者的首选^[78]^。综上所述，对于有严重心肺合并症和不愿接受手术的MPLC患者，消融技术是值得推荐的。然而，为了更全面地评估生存结果，进行较长时间的随访和大样本的队列研究是必要的。

### 6.3 靶向治疗和免疫治疗

靶向治疗大大延长了驱动基因阳性NSCLC患者的OS^[79]^。EGFR-酪氨酸激酶抑制剂（EGFR-tyrosine kinase inhibitors, EGFR-TKIs）作为靶向治疗的代表药物，取得了良好的临床效果。研究^[80]^显示，MPLC具有较高的基因突变率，尤其是EGFR突变，这为MPLC靶向治疗的有效性提供了客观证据。Cheng等^[81]^发现1例sMPLC患者接受了主要病灶的手术切除，在术后12个月接受吉非替尼治疗，其中增大的1个残余病灶达到了完全缓解，其余3个结节保持稳定。该项结果均提示对于无法完全切除的病灶，手术联合靶向治疗是一种有前景的方案。然而，我们也观察到了新的问题。具有驱动基因阳性的病灶并不能代表其他所有病灶，MPLC患者之间及同一患者的不同病灶之间均存在突变差异性。Chen等^[82]^对61个病灶进行基因检测，共检测到77个驱动突变，MPLC患者之间基因改变差异率高达89.7%。此外，有研究者^[83]^对MPLC患者标本进行了全外显子测序，进一步从分子角度证明了肿瘤之间的异质性，认为仅基于一个结节的基因测序来使用靶向治疗是不可靠的，在考虑对多发性肺癌患者进行靶向治疗时，确定每个病灶的突变状态尤为重要。

在免疫治疗中，程序性细胞死亡配体1（programmed cell death ligand 1, PD-L1）被认为是选择免疫检查点抑制剂（immune checkpoint inhibitors, ICIs）治疗患者时最重要的生物标志物^[84]^。Wu等^[85]^对GGNs病变为主的sMPLC患者病例进行了疗效分析，3个周期Pembrolizumab治疗后12个月随访，几乎所有残余GGNs都达到了影像学上的完全缓解。推测免疫治疗可能是治疗MPLC患者的潜在方案。然而，分析接受抗PD-1/PD-L1治疗的肺腺癌队列18例患者的37个同步性GGNs病灶发现，只有8.1%的混合GGNs对治疗有反应，67.6%的GGNs无明显变化，单独使用新辅助PD-1抑制剂可能不是MPLC的最佳治疗策略^[86]^。综上，在部分研究中，运用ICIs显示出了一定疗效，但总体争议较大，其在MPLC中的应用仍面临巨大挑战。

综上所述，手术切除仍是治疗MPLC的首选方案，肺叶切除和亚肺叶切除均可接受，但应避免全肺切除术；放疗和局部消融是治疗MPLC安全有效的手段，对于手术高风险和不愿手术的患者是值得推荐的，且放疗已成为早期不可切除肺癌的首选方案，但二者均缺少随机对照试验和长期的生存分析；此外，靶向治疗和免疫治疗在小样本的回顾性研究中显示出了一定的临床疗效，可作为MPLC有希望的治疗方式，但目前的临床疗效差异较大，不作为主要的治疗手段^[87]^。此外，本文还总结了不同治疗手段的不良反应发生和肺功能情况，见表2。

**表2:** MPLCs不同治疗手段的结果对比

Treatment modality	Authors	Patient group	Pulmonary function	Side effects			Local control status			Survival outcome	
				Grade	Incidence rate		Local control time	Local control rate		Follow-up duration	Survival rate
Surgical intervention	Watanabe T, et al.^[52]^	Sublobar resection: n=76	Sublobar resection has similar survival and local control rates to lobectomy, but preserves more pulmonary function	≥3	17.6%		-	-		5.0 yr	85.0%
	Nomori H, et al.^[53]^	Lobectomy: n=93		≥3	11.8%		5.0 yr	84.0%		5.0 yr	86.0%
		Segmentectomy resection: n=93			9.7%			79.0%			86.0%
Radiation therapy	Nikitas J, et al.^[87]^	Surgery+SABR: n=108; SABR: n=48	Post-radiotherapy, pulmonary function declines in a dose-dependent manner, as evidenced by reductions in FEV_1_ and DLCO; however, the overall data on related pulmonary function are limited	≥3	5.6%		3.0 yr	98.2%		3.0 yr	79.7%
	Dong B, et al.^[67]^	Surgery+SABR: n=73		≥3	2.7%		3.0 yr	86.9%		-	-
	Griffioen GHMJ, et al.^[69]^	Surgery+SABR: n=6; SABR: n=56		≥3	4.8%		2.0 yr	84.0%		2.0 yr	56.0%
Local ablation	Wang Y, et al.^[73]^	MWA: n=46	Pulmonary function may be associated with age, ablation volume, and the number of ablated lesions. There may be a short-term decline in pulmonary function after ablation, but the majority of patients recover to preoperative levels within six months	≥2	23.9%		-	-		2.0 yr	91.3%
	Han X, et al.^[76]^	MWA: n=101		≥3	19.6%		3.0 yr	98.9%		3.0 yr	100%

-: Insufficient data available. SABR: stereotactic ablative radiotherapy; FEV_1_: forced expiratory volume in one second; DLCO: diffusing capacity of the lung for carbon monoxide; MWA: microwave ablation.

## 7 结论与展望

综上所述，随着新技术的助力，MPLC和IM的鉴别诊断取得了快速发展，但两者的鉴别依然是讨论的重点。CHA和NGS的加入使我们更深入地认识了MPLC，同时较好地促进了靶向药物的运用。遗传和环境等因素与MPLC息息相关。在MPLC的后续管理中，手术依然是主要治疗方案，但手术切除的具体范围需要充分收集患者信息，并最好通过多学科讨论决策。SABR和消融治疗对于早期或不可切除的患者是相对安全有效的选择，但需要更多的前瞻性研究来评估生存。MPLC的靶向治疗和免疫治疗在术前和术后环节也取得了一些令人满意的结果。此外，新兴的联合治疗方案也正在探索当中。然而，MPLC的管理仍然具有挑战性，需要进一步深入研究制定个性化的治疗方案。

**Competing interests**

The authors declare that they have no competing interests.

## References

[1] Liu S, Chen Q, Guo L, et al. Incidence and mortality of lung cancer in China, 2008-2012. Chin J Cancer Res, 2018, 30(6): 580-587. doi: 10.21147/j.issn.1000-9604.2018.06.02 30700926 PMC6328502

[2] Siegel RL, Miller KD, Fuchs HE, et al. Cancer statistics, 2022. CA Cancer J Clin, 2022, 72(1): 7-33. doi: 10.3322/caac.21708 35020204

[3] Tie H, Luo J, Shi R, et al. Characteristics and prognosis of synchronous multiple primary lung cancer after surgical treatment: A systematic review and meta-analysis of current evidence. Cancer Med, 2021, 10(2): 507-520. doi: 10.1002/cam4.3614 33300681 PMC7877344

[4] Wang X, Wang M, MacLennan GT, et al. Evidence for common clonal origin of multifocal lung cancers. J Natl Cancer Inst, 2009, 101(8): 560-570. doi: 10.1093/jnci/djp054 19351924

[5] Liao Z, Rivin Del Campo E, Salem A, et al. Optimizing lung cancer radiation treatment worldwide in COVID-19 outbreak. Lung Cancer, 2020, 146: 230-235. doi: 10.1016/j.lungcan.2020.05.029 32585497 PMC7250079

[6] Beyreuther H. Multiplicität von Carcinomen bei einem Fall von sog. “Schneeberger” Lungenkrebs mit Tuberkulose. Virchows Arch Path Anat, 1924, 250(1-2): 230-243. doi: 10.1007/bf01891568

[7] Martini N, Melamed MR. Multiple primary lung cancers. J Thorac Cardiovasc Surg, 1975, 70(4): 606-612. 170482

[8] Wang JF, Zhang T, Ding HL. Research progress in distinguishing methods of simultaneous multiple primary lung cancer and intrapulmonary metastasis. Zhongguo Feiai Zazhi, 2021, 24(5): 365-371. 34034461 10.3779/j.issn.1009-3419.2021.101.12 PMC8174114

[9] Shintani Y, Okami J, Ito H, et al. Clinical features and outcomes of patients with stage I multiple primary lung cancers. Cancer Sci, 2021, 112(5): 1924-1935. doi: 10.1111/cas.14748 33236385 PMC8088915

[10] Chen TF, Xie CY, Rao BY, et al. Surgical treatment to multiple primary lung cancer patients: a systematic review and meta-analysis. BMC Surg, 2019, 19(1): 185. doi: 10.1186/s12893-019-0643-0 31795997 PMC6892192

[11] Ripley RT, McMillan RR, Sima CS, et al. Second primary lung cancers: smokers versus nonsmokers after resection of stage I lung adenocarcinoma. Ann Thorac Surg, 2014, 98(3): 968-974. doi: 10.1016/j.athoracsur.2014.04.098 25038021 PMC4410355

[12] Nicholson AG, Torkko K, Viola P, et al. Interobserver variation among pathologists and refinement of criteria in distinguishing separate primary tumors from intrapulmonary metastases in lung. J Thorac Oncol, 2018, 13(2): 205-217. doi: 10.1016/j.jtho.2017.10.019 29127023 PMC6276791

[13] Moore DA, Sereno M, Das M, et al. In situ growth in early lung adenocarcinoma may represent precursor growth or invasive clone outgrowth-a clinically relevant distinction. Mod Pathol, 2019, 32(8): 1095-1105. doi: 10.1038/s41379-019-0257-1 30932019

[14] Rami-Porta R, Asamura H, Travis WD, et al. Lung cancer - major changes in the American Joint Committee on Cancer eighth edition cancer staging manual. CA Cancer J Clin, 2017, 67(2): 138-155. doi: 10.3322/caac.21390 28140453

[15] Yang SR, Chang JC, Leduc C, et al. Invasive mucinous adenocarcinomas with spatially separate lung lesions: analysis of clonal relationship by comparative molecular profiling. J Thorac Oncol, 2021, 16(7): 1188-1199. doi: 10.1016/j.jtho.2021.03.023 33839364 PMC8240964

[16] Wang X, Liu H, Zhai D, et al. Multiple primary lung tumors of different pathological types including squamous cell carcinoma, adenocarcinoma, and mixed squamous cell and glandular papilloma: a case report. Onco Targets Ther, 2022, 15: 13-19. doi: 10.2147/OTT.S344086 35023930 PMC8747777

[17] Detterbeck FC, Franklin WA, Nicholson AG, et al. The IASLC lung cancer staging project: background data and proposed criteria to distinguish separate primary lung cancers from metastatic foci in patients with two lung tumors in the forthcoming eighth edition of the TNM classification for lung cancer. J Thorac Oncol, 2016, 11(5): 651-665. doi: 10.1016/j.jtho.2016.01.025 26944304

[18] Chang JC, Alex D, Bott M, et al. Comprehensive next-generation sequencing unambiguously distinguishes separate primary lung carcinomas from intrapulmonary metastases: comparison with standard histopathologic approach. Clin Cancer Res, 2019, 25(23): 7113-7125. doi: 10.1158/1078-0432.CCR-19-1700 31471310 PMC7713586

[19] Girard N, Ostrovnaya I, Lau C, et al. Genomic and mutational profiling to assess clonal relationships between multiple non-small cell lung cancers. Clin Cancer Res, 2009, 15(16): 5184-5190. doi: 10.1158/1078-0432.CCR-09-0594 19671847 PMC2892178

[20] Huang M, Xu Q, Zhou M, et al. Distinguishing multiple primary lung cancers from intrapulmonary metastasis using CT-based radiomics. Eur J Radiol, 2023, 160: 110671. doi: 10.1016/j.ejrad.2022.110671 36739831

[21] Zhou LN, Wu N, Zhao SJ, et al. HRCT features differentiate synchronous multiple primary lung adenocarcinomas from intrapulmonary metastases. Zhonghua Zhongliu Zazhi, 2020, 42(6): 449-455. 32575939 10.3760/cma.j.cn112152-20200227-00126

[22] Li R, Li X, Xue R, et al. Early metastasis detected in patients with multifocal pulmonary ground-glass opacities (GGOs). Thorax, 2018, 73(3): 290-292. doi: 10.1136/thoraxjnl-2017-210169 29056599 PMC5870446

[23] Liu Y, Tang Y, Xue Z, et al. SUVmax ratio on PET/CT may differentiate between lung metastases and synchronous multiple primary lung cancer. Acad Radiol, 2020, 27(5): 618-623. doi: 10.1016/j.acra.2019.07.001 31787567

[24] Suh YJ, Lee HJ, Sung P, et al. A novel algorithm to differentiate between multiple primary lung cancers and intrapulmonary metastasis in multiple lung cancers with multiple pulmonary sites of involvement. J Thorac Oncol, 2020, 15(2): 203-215. doi: 10.1016/j.jtho.2019.09.221 31634666

[25] Wani NA, Kumar R, Bedi J. DeepXplainer: An interpretable deep learning based approach for lung cancer detection using explainable artificial intelligence. Comput Methods Programs Biomed, 2023, 243: 107879. doi: 10.1016/j.cmpb.2023.107879 37897989

[26] Li X, Hu B, Li H, et al. Application of artificial intelligence in the diagnosis of multiple primary lung cancer. Thorac Cancer, 2019, 10(11): 2168-2174. doi: 10.1111/1759-7714.13185 31529684 PMC6825907

[27] Hu C, Zhao L, Liu W, et al. Genomic profiles and their associations with TMB, PD-L 1 expression, and immune cell infiltration landscapes in synchronous multiple primary lung cancers. J Immunother Cancer, 2021, 9(12): e003773. doi: 10.1136/jitc-2021-003773 34887263 PMC8663088

[28] Weinberg BA, Gowen K, Lee TK, et al. Comprehensive genomic profiling aids in distinguishing metastatic recurrence from second primary cancers. Oncologist, 2017, 22(2): 152-157. doi: 10.1634/theoncologist.2015-0511 28193735 PMC5330711

[29] Mansuet-Lupo A, Barritault M, Alifano M, et al. Proposal for a combined histomolecular algorithm to distinguish multiple primary adenocarcinomas from intrapulmonary metastasis in patients with multiple lung tumors. J Thorac Oncol, 2019, 14(5): 844-856. doi: 10.1016/j.jtho.2019.01.017 30721797

[30] Tian H, Bai G, Yang Z, et al. Multiple primary lung cancer: Updates of clinical management and genomic features. Front Oncol, 2023, 13: 1034752. doi: 10.3389/fonc.2023.1034752 36910635 PMC9993658

[31] Liu Y, Zhang J, Li L, et al. Genomic heterogeneity of multiple synchronous lung cancer. Nat Commun, 2016, 7: 13200. doi: 10.1038/ncomms13200 27767028 PMC5078731

[32] Li Y, Li X, Li H, et al. Genomic characterisation of pulmonary subsolid nodules: mutational landscape and radiological features. Eur Respir J, 2020, 55(2): 1-4. doi: 10.1183/13993003.01409-2019 31699841

[33] Liu J, Mao G, Li Y, et al. Targeted deep sequencing helps distinguish independent primary tumors from intrapulmonary metastasis for lung cancer diagnosis. J Cancer Res Clin Oncol, 2020, 146(9): 2359-2367. doi: 10.1007/s00432-020-03227-5 32333141 PMC11804704

[34] Guo W, Zhou B, Bie F, et al. Single-cell RNA sequencing analysis reveals transcriptional heterogeneity of multiple primary lung cancer. Clin Transl Med, 2023, 13(10): e1453. doi: 10.1002/ctm2.1453 37846760 PMC10580343

[35] Aredo JV, Luo SJ, Gardner RM, et al. Tobacco smoking and risk of second primary lung cancer. J Thorac Oncol, 2021, 16(6): 968-979. doi: 10.1016/j.jtho.2021.02.024 33722709 PMC8159872

[36] Bhaskarla A, Tang PC, Mashtare T, et al. Analysis of second primary lung cancers in the SEER database. J Surg Res, 2010, 162(1): 1-6. doi: 10.1016/j.jss.2009.12.030 20400118

[37] Li C, Wang Y, Su K, et al. Presentation of EGFR mutations in 162 family probands with multiple primary lung cancer. Transl Lung Cancer Res, 2021, 10(4): 1734-1746. doi: 10.21037/tlcr-20-1001 34012789 PMC8107753

[38] Chen Q, Gao J, Yu H, et al. An emerging role of microplastics in the etiology of lung ground glass nodules. Environ Sci Eur, 2021, 16: 1-5. doi: 10.1186/s12302-022-00605-3

[39] Chen Y, Li G, Lei Y, et al. Lung cancer family history and exposure to occupational/domestic coal combustion contribute to variations in clinicopathologic features and gene fusion patterns in non-small cell lung cancer. Thorac Cancer, 2019, 10(4): 695-707. doi: 10.1111/1759-7714.12987 30775858 PMC6449330

[40] Wei S, Chen M, Chen N, et al. Feasibility and safety of robot-assisted thoracic surgery for lung lobectomy in patients with non-small cell lung cancer: a systematic review and meta-analysis. World J Surg Oncol, 2017, 15(1): 98. doi: 10.1186/s12957-017-1168-6 28482928 PMC5422947

[41] Chen D, Du M, Yang T. Uniportal video-assisted thoracoscopic lobectomy for lung cancer. J Thorac Dis, 2016, 8(7): 1830-1833. doi: 10.21037/jtd.2016.05.58 27499976 PMC4958878

[42] Zuin A, Andriolo LG, Marulli G, et al. Is lobectomy really more effective than sublobar resection in the surgical treatment of second primary lung cancer? Eur J Cardiothorac Surg, 2013, 44(2): e120-e125. doi: 10.1093/ejcts/ezt219 23657547

[43] Ginsberg R, Rubinstein LV. Randomized trial of lobectomy versus limited resection for T1 N0 non-small cell lung cancer. Lung Cancer Study Group. Ann Thorac Surg, 1995, 60(3): 615-622. doi: 10.1016/0003-4975(95)00537-u 7677489

[44] Aziz T, Saad RA, Glasser J, et al. The management of second primary lung cancers. A single centre experience in 15 years. Eur J Cardio-Thorac, 2002, 21(3): 527-533. doi: 10.1016/s1010-7940(02)00024-6 11888775

[45] Khullar OV, Liu Y, Gillespie T, et al. Survival after sublobar resection versus lobectomy for clinical stage IA lung cancer: an analysis from the national cancer data base. J Thorac Oncol, 2015, 10(11): 1625-1633. doi: 10.1097/JTO.0000000000000664 26352534 PMC5798611

[46] Shimada Y, Saji H, Otani K, et al. Survival of a surgical series of lung cancer patients with synchronous multiple ground-glass opacities, and the management of their residual lesions. Lung Cancer, 2015, 88(2): 174-180. doi: 10.1016/j.lungcan.2015.02.016 25758554

[47] Kikunaga S, Fujimori S, Suzuki S, et al. The strategy and outcome of three-port thoracoscopic surgery for synchronous and metachronous multiple lung cancer. Kyobu Geka, 2021, 74(1): 33-39. 33550317

[48] Ishikawa Y, Nakayama H, Ito H, et al. Surgical treatment for synchronous primary lung adenocarcinomas. Ann Thorac Surg, 2014, 98(6): 1983-1988. doi: 10.1016/j.athoracsur.2014.07.006 25443005

[49] Kocaturk CI, Gunluoglu MZ, Cansever L, et al. Survival and prognostic factors in surgically resected synchronous multiple primary lung cancers. Eur J Cardiothorac Surg, 2011, 39(2): 160-166. doi: 10.1016/j.ejcts.2010.05.037 20650645

[50] Jung EJ, Lee JH, Jeon K, et al. Treatment outcomes for patients with synchronous multiple primary non-small cell lung cancer. Lung Cancer, 2011, 73(2): 237-242. doi: 10.1016/j.lungcan.2010.11.008 21145616

[51] Tsunezuka Y, Fujimori H, Tanaka N. Surgical treatment to bilateral multiple primary lung cancer patients. Kyobu Geka, 2021, 74(1): 49-53. 33550319

[52] Watanabe T, Tanahashi M, Suzuki E, et al. Surgical treatment for synchronous multiple primary lung cancer: Is it possible to achieve both curability and preservation of the pulmonary function? Thorac Cancer, 2021, 12(22): 2996-3004. doi: 10.1111/1759-7714.14164 34590424 PMC8590900

[53] Nomori H, Yamazaki I, Machida Y, et al. Lobectomy versus segmentectomy: a propensity score-matched comparison of postoperative complications, pulmonary function and prognosis. Interact Cardiovasc Thorac Surg, 2022, 34(1): 57-65. doi: 10.1093/icvts/ivab212 34999814 PMC8743134

[54] Nie Y, Wang X, Yang F, et al. Surgical prognosis of synchronous multiple primary lung cancer: systematic review and meta-analysis. Clin Lung Cancer, 2021, 22(4): 341-350.e3. doi: 10.1016/j.cllc.2020.10.022 33243621

[55] Yang H, Sun Y, Yao F, et al. Surgical therapy for bilateral multiple primary lung cancer. Ann Thorac Surg, 2016, 101(3): 1145-1152. doi: 10.1016/j.athoracsur.2015.09.028 26602007

[56] Bae MK, Byun CS, Lee CY, et al. The role of surgical treatment in second primary lung cancer. Ann Thorac Surg, 2011, 92(1): 256-262. doi: 10.1016/j.athoracsur.2011.02.034 21600562

[57] Jiang L, He J, Shi X, et al. Prognosis of synchronous and metachronous multiple primary lung cancers: systematic review and meta-analysis. Lung Cancer, 2015, 87(3): 303-310. doi: 10.1016/j.lungcan.2014.12.013 25617985

[58] Cho S, Yang H, Kim K, et al. Pathology and prognosis of persistent stable pure ground-glass opacity nodules after surgical resection. Ann Thorac Surg, 2013, 96(4): 1190-1195. doi: 10.1016/j.athoracsur.2013.05.062 23968760

[59] Kim H, Choi YS, Kim J, et al. Management of multiple pure ground-glass opacity lesions in patients with bronchioloalveolar carcinoma. J Thorac Oncol, 2010, 5(2): 206-210. doi: 10.1097/JTO.0b013e3181c422be 19901852

[60] Shimada Y, Maehara S, Kudo Y, et al. Profiles of lung adenocarcinoma with multiple ground-glass opacities and the fate of residual lesions. Ann Thorac Surg, 2020, 109(6): 1722-1730. doi: 10.1016/j.athoracsur.2019.12.062 32057816

[61] Hattori A, Takamochi K, Oh S, et al. Prognostic classification of multiple primary lung cancers based on a ground-glass opacity component. Ann Thorac Surg, 2020, 109(2): 420-427. doi: 10.1016/j.athoracsur.2019.09.008 31593656

[62] Vinod SK, Hau E. Radiotherapy treatment for lung cancer: Current status and future directions. Respirology, 2020, 25 Suppl 2: 61-71. doi: 10.1111/resp.13870 32516852

[63] Ball D, Mai GT, Vinod S, et al. Stereotactic ablative radiotherapy versus standard radiotherapy in stage 1 non-small-cell lung cancer (TROG 09.02 CHISEL): a phase 3, open-label, randomised controlled trial. Lancet Oncol, 2019, 20(4): 494-503. doi: 10.1016/S1470-2045(18)30896-9 30770291

[64] Haque W, Verma V, Polamraju P, et al. Stereotactic body radiation therapy versus conventionally fractionated radiation therapy for early stage non-small cell lung cancer. Radiother Oncol, 2018, 129(2): 264-269. doi: 10.1016/j.radonc.2018.07.008 30031630

[65] Chang JY, Mehran RJ, Feng L, et al. Stereotactic ablative radiotherapy for operable stage I non-small-cell lung cancer (revised STARS): long-term results of a single-arm, prospective trial with prespecified comparison to surgery. Lancet Oncol, 2021, 22(10): 1448-1457. doi: 10.1016/S1470-2045(21)00401-0 34529930 PMC8521627

[66] Park K, Vansteenkiste J, Lee KH, et al. Pan-Asian adapted ESMO Clinical Practice Guidelines for the management of patients with locally-advanced unresectable non-small-cell lung cancer: a KSMO-ESMO initiative endorsed by CSCO, ISMPO, JSMO, MOS, SSO and TOS. Ann Oncol, 2020, 31(2): 191-201. doi: 10.1016/j.annonc.2019.10.026 31959336

[67] Dong B, Chen R, Zhu X, et al. Comparison of stereotactic body radiation therapy versus surgery for multiple primary lung cancers after prior radical resection: A multicenter retrospective study. Clin Transl Radiat Oncol, 2023, 40: 100601. doi: 10.1016/j.ctro.2023.100601 36936471 PMC10020093

[68] Shintani T, Masago K, Takayama K, et al. Stereotactic body radiotherapy for synchronous primary lung cancer: clinical outcome of 18 cases. Clin Lung Cancer, 2015, 16(5): e91-e96. doi: 10.1016/j.cllc.2014.12.011 25659504

[69] Griffioen GHMJ, Lagerwaard FJ, Haasbeek CJA, et al. Treatment of multiple primary lung cancers using stereotactic radiotherapy, either with or without surgery. Radiother Oncol, 2013, 107(3): 403-408. doi: 10.1016/j.radonc.2013.04.026 23746675

[70] Chang J, Lin SH, Dong W, et al. Stereotactic ablative radiotherapy with or without immunotherapy for early-stage or isolated lung parenchymal recurrent node-negative non-small-cell lung cancer: an open-label, randomised, phase 2 trial. Lancet, 2023, 402(10405): 871-881. doi: 10.1016/S0140-6736(23)01384-3 37478883 PMC10529504

[71] Paez-Carpio A, Gomez FM, Isus Olive G, et al. Image-guided percutaneous ablation for the treatment of lung malignancies: current state of the art. Insights Imaging, 2021, 12(1): 57. doi: 10.1186/s13244-021-00997-5 33914187 PMC8085189

[72] Ye X, Fan WJ, Wang ZM, et al. Expert consensus for thermal ablation of pulmonary subsolid nodules (2021 edition). Zhongguo Feiai Zazhi, 2021, 24(5): 305-322. 33896152 10.3779/j.issn.1009-3419.2021.101.14 PMC8174112

[73] Wang Y, Liu B, Cao P, et al. Comparison between computed tomography-guided percutaneous microwave ablation and thoracoscopic lobectomy for stage I non-small cell lung cancer. Thorac Cancer, 2018, 9(11): 1376-1382. doi: 10.1111/1759-7714.12842 30152596 PMC6209786

[74] Huang G, Yang X, Li W, et al. A feasibility and safety study of computed tomography-guided percutaneous microwave ablation: a novel therapy for multiple synchronous ground-glass opacities of the lung. Int J Hyperthermia, 2020, 37(1): 414-422. doi: 10.1080/02656736.2020.1756467 32347133

[75] Bao F, Yu F, Wang R, et al. Electromagnetic bronchoscopy guided microwave ablation for early stage lung cancer presenting as ground glass nodule. Transl Lung Cancer Res, 2021, 10(9): 3759-3770. doi: 10.21037/tlcr-21-474 34733626 PMC8512468

[76] Han X, Wei Z, Zhao Z, et al. Cost and effectiveness of microwave ablation versus video-assisted thoracoscopic surgical resection for ground-glass nodule lung adenocarcinoma. Front Oncol, 2022, 12: 962630. doi: 10.3389/fonc.2022.962630 36276106 PMC9581221

[77] de Koning HJ, van der Aalst CM, de Jong PA, et al. Reduced lung-cancer mortality with volume CT screening in a randomized trial. N Engl J Med, 2020, 382(6): 503-513. doi: 10.1056/NEJMoa1911793 31995683

[78] Zhang Y, Fu F, Chen H. Management of ground-glass opacities in the lung cancer spectrum. Ann Thorac Surg, 2020, 110(6): 1796-1804. doi: 10.1016/j.athoracsur.2020.04.094 32525031

[79] Wang M, Herbst RS, Boshoff C. Toward personalized treatment approaches for non-small-cell lung cancer. Nat Med, 2021, 27(8): 1345-1356. doi: 10.1038/s41591-021-01450-2 34385702

[80] Liu M, He WX, Song N, et al. Discrepancy of epidermal growth factor receptor mutation in lung adenocarcinoma presenting as multiple ground-glass opacities. Eur J Cardiothorac Surg, 2016, 50(5): 909-913. doi: 10.1093/ejcts/ezw113 27032467

[81] Cheng B, Deng H, Zhao Y, et al. Management for residual ground-glass opacity lesions after resection of main tumor in multifocal lung cancer: a case report and literature review. Cancer Manag Res, 2021, 13: 977-985. doi: 10.2147/CMAR.S290830 33568943 PMC7868271

[82] Chen K, Chen W, Cai J, et al. Favorable prognosis and high discrepancy of genetic features in surgical patients with multiple primary lung cancers. J Thorac Cardiovasc Surg, 2018, 155(1): 371-379.e1. doi: 10.1016/j.jtcvs.2017.08.141 29092754

[83] Teng F, Xu J, Wang J, et al. Correlation between gene mutation status and clinicopathologic features in early multiple primary lung cancer. Front Oncol, 2023, 13: 1110259. doi: 10.3389/fonc.2023.1110259 37124493 PMC10130385

[84] Reck M, Rodriguez-Abreu D, Robinson AG, et al. Pembrolizumab versus chemotherapy for PD-L1-positive non-small-cell lung cancer. N Engl J Med, 2016, 375(19): 1823-1833. doi: 10.1056/NEJMoa1606774 27718847

[85] Wu S, Li D, Chen J, et al. Tailing effect of PD-1 antibody results in the eradication of unresectable multiple primary lung cancer presenting as ground-glass opacities: a case report. Ann Palliat Med, 2021, 10(1): 778-784. doi: 10.21037/apm-20-2132 33545799

[86] Wu F, Li W, Zhao W, et al. Synchronous ground‐glass nodules showed limited response to anti‐PD‐1/PD‐L 1 therapy in patients with advanced lung adenocarcinoma. Clin Transl Med, 2023, 10(3): e149. doi: 10.1002/ctm2.149

[87] Nikitas J, DeWees T, Rehman S, et al. Stereotactic body radiotherapy for early-stage multiple primary lung cancers. Clin Lung Cancer, 2019, 20(2): 107-116. doi: 10.1016/j.cllc.2018.10.010 30477740 PMC6387838

